# The Axon Guidance Receptor Gene *ROBO1* Is a Candidate Gene for Developmental Dyslexia 

**DOI:** 10.1371/journal.pgen.0010050

**Published:** 2005-10-28

**Authors:** Katariina Hannula-Jouppi, Nina Kaminen-Ahola, Mikko Taipale, Ranja Eklund, Jaana Nopola-Hemmi, Helena Kääriäinen, Juha Kere

**Affiliations:** 1 Department of Medical Genetics, University of Helsinki, Finland; 2 European Molecular Biology Laboratory, Gene Expression Programme, Heidelberg, Germany; 3 Department of Pediatrics, Jorvi Hospital, Espoo, Finland; 4 Department of Medical Genetics, The Family Federation of Finland, Helsinki, Finland; 5 Department of Medical Genetics, University of Turku, Turku, Finland; 6 Department of Biosciences at Novum and Clinical Research Centre, Karolinska Institutet, Stockholm, Sweden; University of Oxford, United Kingdom

## Abstract

Dyslexia, or specific reading disability, is the most common learning disorder with a complex, partially genetic basis, but its biochemical mechanisms remain poorly understood. A locus on Chromosome 3, *DYX5,* has been linked to dyslexia in one large family and speech-sound disorder in a subset of small families. We found that the axon guidance receptor gene *ROBO1,* orthologous to the *Drosophila roundabout* gene, is disrupted by a chromosome translocation in a dyslexic individual. In a large pedigree with 21 dyslexic individuals genetically linked to a specific haplotype of *ROBO1* (not found in any other chromosomes in our samples), the expression of *ROBO1* from this haplotype was absent or attenuated in affected individuals. Sequencing of *ROBO1* in apes revealed multiple coding differences, and the selection pressure was significantly different between the human, chimpanzee, and gorilla branch as compared to orangutan. We also identified novel exons and splice variants of *ROBO1* that may explain the apparent phenotypic differences between human and mouse in heterozygous loss of *ROBO1.* We conclude that dyslexia may be caused by partial haplo-insufficiency for *ROBO1* in rare families. Thus, our data suggest that a slight disturbance in neuronal axon crossing across the midline between brain hemispheres, dendrite guidance, or another function of *ROBO1* may manifest as a specific reading disability in humans.

## Synopsis

Dyslexia, or specific reading disability, is a common learning disorder with a complex, partially genetic basis. A number of chromosomal regions harboring genes involved in dyslexia have been identified, and in this study the authors describe a candidate gene from one such locus, called *DYX5,* on Chromosome 3. The authors show that an axon guidance receptor gene, *ROBO1,* is disrupted by a chromosomal translocation in one dyslexic individual; furthermore, this study shows that the expression of *ROBO1* is reduced on chromosomes from dyslexics in a large pedigree in which dyslexia has been linked to *DYX5. ROBO1* has a role in regulating axon crossing across the midline between brain hemispheres and guidance of neuronal dendrites. As suggested by these findings, dyslexia may be caused in rare families by a small change in the expression of *ROBO1,* such as loss of one functional copy. Thus, *ROBO1* is a candidate for a dyslexia susceptibility gene.

## Introduction

Dyslexia refers to a difficulty in reading and writing despite normal intelligence, senses, and adequate education. The primary difficulty lies in phonological processing, rapid naming, and the recognition of phonemes [1]. Dyslexia is a common disorder, affecting 3% to 10% of the population [2]. Familial occurrence has been reported and twin and family studies indicate a strong genetic component in its etiology [3]. So far, genome-wide screens have linked dyslexia to loci on Chromosomes 1p34–36 *(DYX8),* 2p16-p15 *(DYX3),* 3p12-q13 *(DYX5),* 6p21.3 *(DYX2),* 11p15.5 *(DYX7),* 15q21 *(DYX1),* 18p11.2 *(DYX6),* Xq27.3 *(DYX9),* and 7q32 (http://www.ncbi.nlm.nih.gov/omim)[4–12]. The first dyslexia candidate susceptibility gene, *DYX1C1* at 15q21, was recently identified, and a region corresponding to *DYX2* has been narrowed down to a small number of candidate genes [12–16].

We ascertained earlier a large four-generation family including 21dyslexic individuals available to the study with severe dyslexia segregating in a dominant fashion, and mapped the susceptibility gene by a genome-wide scan to a region on Chromosome 3 that was named the *DYX5* locus [17]. A 20-cM long haplotype was shared between 19 to 21 dyslexic individuals, giving statistically significant support for the mapping of the gene, but insufficient resolution to identify the gene [17]. Recently, speech-sound disorder was studied in a large set of 77 families from the United States, with results supporting association of this disorder to the *DYX5* locus in the majority of families [18]. Phonological processing was the common phenotypic component found deficient in both studies [17–19].

We report here the localization of a translocation in an individual with dyslexia and a translocation t(3;8)(p12;q11) to the *DYX5* region and specifically to the first intron of *ROBO1. ROBO1* is a neuronal axon guidance receptor gene involved in brain development, and thus an attractive candidate gene for dyslexia susceptibility [20–22]. Furthermore, in the large pedigree with 19 dyslexic individuals genetically linked to *DYX5* and more specifically, a specific and rare haplotype of *ROBO1,* the expression of *ROBO1* from this haplotype was absent or attenuated in affected individuals. We conclude that dyslexia may be caused by partial haplo-insufficiency for *ROBO1.* In addition, we searched for novel exons and splice variants of *ROBO1* that may help in understanding the apparent phenotypic discrepancy between heterozygous *Dutt1*and *Robo1* knockout mice that develop lung cancers and lymphomas and humans whose developmental phenotypic consequence appears to be dyslexia.

## Results

**Figure 1:**
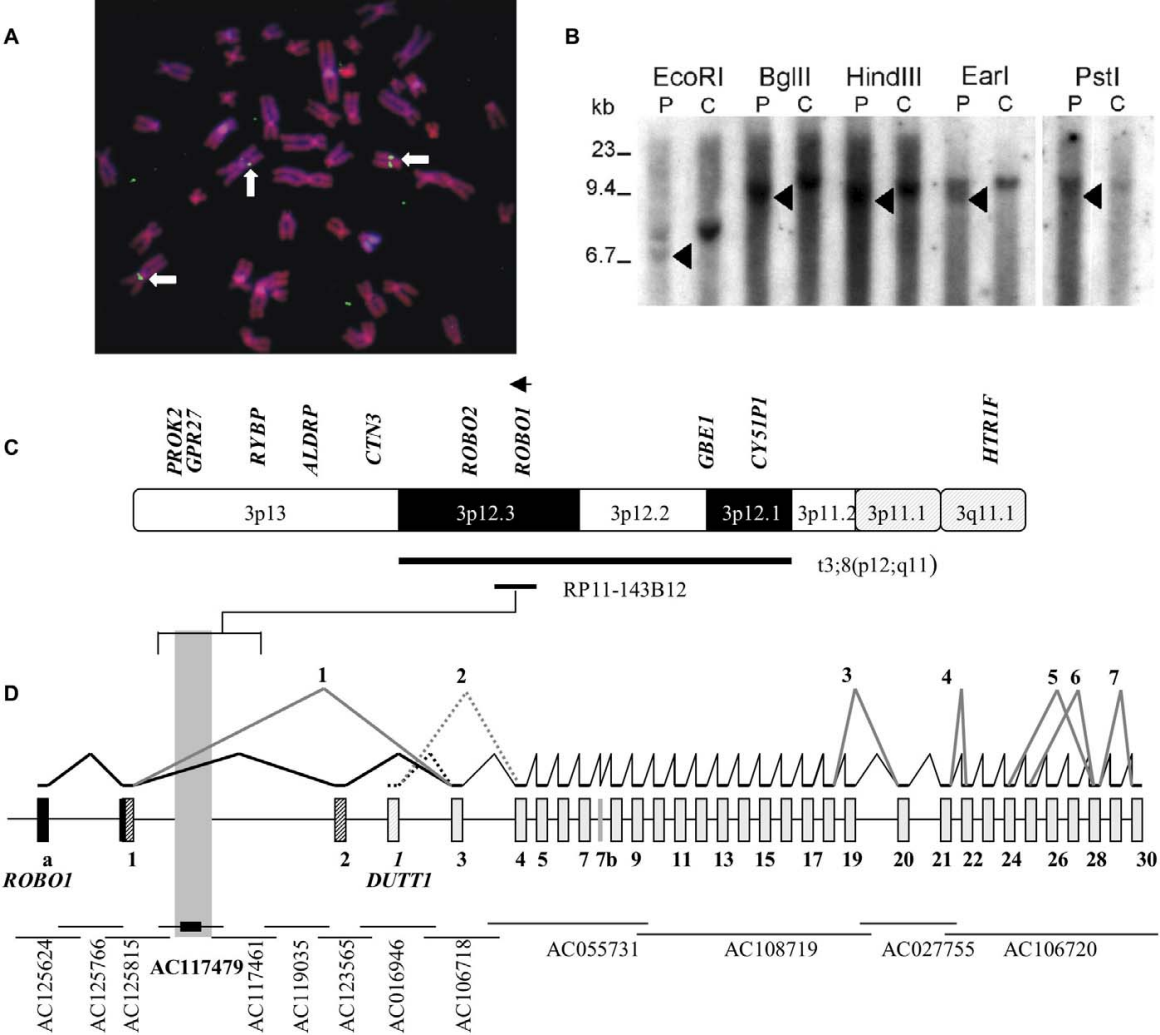
Delineation of Translocation Breakpoint Region and *ROBO1* Structure (A) Fluorescence in situ hybridization with BAC clone RP11-143B12 as a probe, showing hybridization signals in Chromosome 3 (upward arrow), der(3), and der(8) (horizontal arrows). (B) Southern hybridization with a probe derived from RP11-143B12 shows genomic rearrangements (arrowheads) with five restriction enzymes in translocation patient (P) compared to the control sample. (C) A gene map of Chromosome 3p13-3q11.1 showing the cytogenetic localization of the translocation breakpoint (black bar). An arrow indicates the direction of *ROBO1* transcription. Localization of the translocation breakpoint (square bracket) to BACs AC117479 and AC117461. (D) Splice variants and exon structure of *ROBO1* (exons numbered from 1–30). Novel exons a and 7b and additional sequence to exon 1 are indicated in solid black. Exons unique to *ROBO1* (hatched black) and *DUTT1* (hatched grey) and common to both *ROBO1* and *DUTT1* (solid grey) are indicated. Corresponding BACs to exons are shown below. The translocation disrupting *ROBO1* between exons 1 and 2 in AC117479 is shown by vertical grey bar. Dotted lines indicate *DUTT1* variants. Novel splice variants are shown by grey lines and numbered (1), exclusion of exon 2 (89–169 of AF040990) (2), exclusion of *DUTT1* exon 2 (1019–1345 of Z95705) (3), exclusion of exon 19 (2813–2829 of AF040990) (4), initial 165 bp of exon 22 (3037–3201 of AF040990) (5), 905 bp from exons 24–28 (3603–4508 of AF040990) (6), 878 bp from exons 25–28 (3641–4528 of AF040990) and (7), exclusion of exon 29 (4745–4939 of AF040990).

We identified a patient who was diagnosed with both dyslexia and a translocation t(3;8)(p12;q11) involving the *DYX5* region. He came to our attention initially because of infertility. He had three siblings, one of whom was also diagnosed with dyslexia, but two were of subnormal intelligence and thus undefined for the dyslexia phenotype. Detailed phenotypic comparison was not possible, because the family members were not available for retesting. The index patient was the only translocation carrier among the siblings and likely to possess a de novo translocation (see Materials and Methods for details of the family). In spite of the discrepancy between the translocation status and apparent concordance for dyslexia between two siblings, we decided to map the translocation breakpoint in the hope of gaining insight to a possible candidate gene for further evaluation in the genetically informative large family [17,19]. We used fluorescence in situ hybridization to narrow down the breakpoint until a probe bacterial artificial chromosome (BAC) clone RP11-143B12 hybridized to both der(3) chromosomes as well as the normal Chromosome 3 (Figure 1A). The genomic sequence of clone RP11-143B12 corresponds to a Chromosome 3 scaffold sequence on the Celera public database (http://public.celera.com/cds/login.cfm) and to BACs AC117479 (43316–43873 base pairs [bp]) and AC117461 (50209–50745 bp) in the National Center for Biotechnology database (http://www.ncbi.nlm.nih.gov) (Figure 1C). Further identification of the breakpoint was conducted by Southern hybridization with PCR-amplified non-repetitive genomic DNA fragment probes from the clone. A 971-bp probe revealed DNA rearrangements, refining the breakpoint to a 4.7-kilobase (kb) interval on the Celera scaffold sequence or a 4.0-kb interval corresponding to nucleotides 57269–61233 of BAC AC117479 (Figure 1B and 1D). Surprisingly, the translocation breakpoint localized within the ortholog of the *Drosophila roundabout (robo)* gene, *ROBO1,* alternatively spliced and also named *DUTT1* (Deleted in U Twenty Twenty), disrupts *ROBO1* between exons 1 and 2.

**Figure 2:**
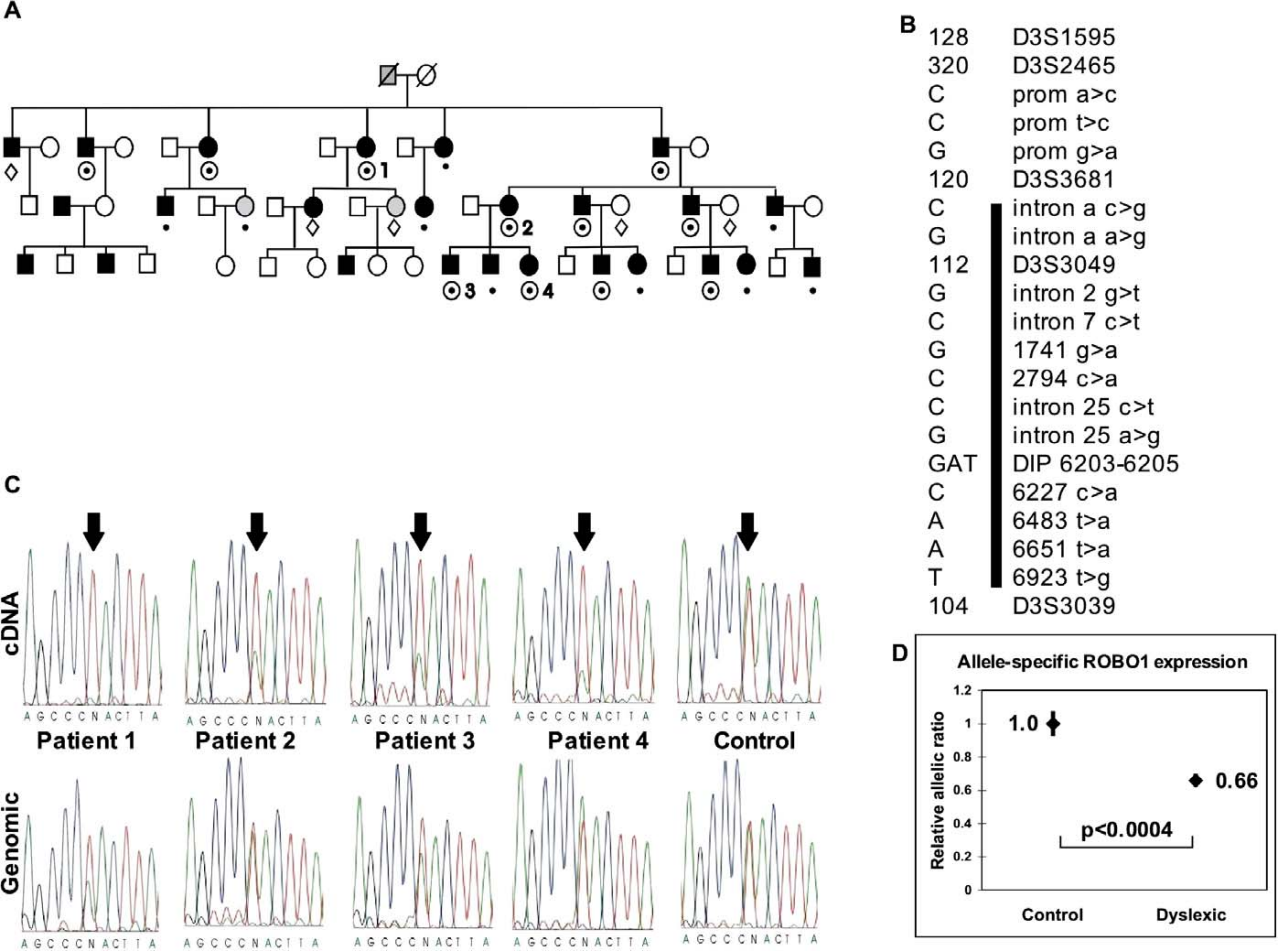
Analysis of *ROBO1* in the Large Family Linked to *DYX5* (A) An abridged pedigree of the family linked to *DYX5* [17,19]. Numbers refer to samples studied for *ROBO1* expression (C and D). A dot indicates carriers of the dyslexia-linked haplotype [17] and circled dots indicate individuals genotyped for all markers (B). Diamonds denote individuals genotyped for all markers, but not sharing the haplotype. Affected individuals are shaded black and unverified dyslectics are shaded gray. (B) Markers (right) and alleles (left) that define the haplotype linked to dyslexia (A). The bar indicates the extent of the *ROBO1* haplotype carried by patients marked with a dot (A). (C) Sequencing of cDNA reveals absent or attenuated expression (*p* < 0.017 for all measurements) of the *ROBO1* allele (SNP 6483A > T indicated by arrows) encoded by the dyslexia-linked haplotype as compared to genomic sequence. In the control, both alleles show equal allelic ratios in genomic and cDNA. Patient numbers refer to (A). (D) Attenuation of *ROBO1* mRNA expression from the dyslexia-associated allele. Allelic expression of *ROBO1* was assessed by sequencing the SNP 6483 (A/T) as in (C). Allelic ratios were assessed by five to six replicated sequencing tracings in four controls (21 data points) and four dyslexic individuals (24 data points). The results are expressed as the mRNA level of the dyslexia-associated allele as compared to the corresponding allele mRNA level in controls. Data are shown as mean ± 1 standard error of the mean (bars). Significance was assessed by two-tailed *t* test.

Because of its known function in neuronal axon guidance in the developing brain, *ROBO1* was a plausible candidate gene for dyslexia susceptibility [20–22]. We sequenced its exons, splice sites, 1 kb of *ROBO1* promoter region upstream of exon 1, and the extended 3′ UTR region of *ROBO1* variant 2 from the genomic DNA of initially one dyslexic individual and his parents (father dyslexic, mother unaffected) from the large linkage family (Figure 2A). All exons were also sequenced from the cDNA of another dyslexic individual from the extended family. Comparison of the sequences to *ROBO1*and *ROBO1* variant 2 sequences revealed altogether seven sequence variations, two of them previously known [23]. All of the observed changes were confirmed in three additional pedigree members (dyslexic father, son, and unaffected mother) by sequencing. Dyslexic individuals had two silent single nucleotide polymorphisms (SNPs) in *ROBO1* exons 12 and 18 (1741G > A, 2794C > A; numbering according to *ROBO1*), an exonic 3-bp deletion and insertion polymorphism (DIP6203–6205; numbering for *ROBO1* variant 2), four SNPs in 3′ UTR (UTR, 6227C > A, 6483T > A, 6651T > A, 6923T > G; numbering for *ROBO1* variant 2), and four intronic SNPs (intron 2: 59567 and intron 7: 1451; numbering for BAC RP11-588D3; intron 25: 16181 and 16198; numbering for BAC RP11-26M20) (Figure 2B).

Genotyping of the exonic SNPs in ten additional family members and two unrelated non-dyslexic individuals confirmed that a specific SNP haplotype segregated with dyslexia, consistent with the previously observed linkage, but revealed that none of the polymorphisms was uniquely observed only in dyslexic individuals. For example, the DIP6203–6205*GAT+ allele had an allele frequency of 22% (94/434 among healthy control participants' chromosomes) and did not show significant association to dyslexia in our replication sample set of 96 dyslexic individuals from other families. None of the four observed intronic SNPs produced alternative splice variants. Because the *ROBO1* gene spans about 990 kb of genomic DNA and contains altogether over 2,200 intronic SNPs (according to the NCBI SNP database), their exhaustive listing in our family members was impractical. In accordance with our previous genome scan on dyslexia in Finland, suggesting that *DYX5* locus is not involved in most families [9], the same haplotype as in this large family was not observed in other, unrelated families (unpublished data).

The silent and 3′ UTR SNPs provided assays to study the transcription of *ROBO1.* Our rationale was to measure whether both alleles of *ROBO1* were equally transcribed in dyslexic individuals segregating the dominant susceptibility haplotype. Comparison of genomic and cDNA samples from four dyslexic individuals showed that *ROBO1* mRNA was only weakly or not at all transcribed from the allele that segregated with dyslexia (Figure 2C), whereas in non-readers' lymphocytes, as well as control brain RNA, biallelic expression was consistently observed. Of note, there was considerable variation between individuals, suggesting that the regulation of expression is complex. The SNP 2794C > A was heterozygous in one patient, 6483T > A in four, 6651T > A in four, and 6923T > G in one, and combining all results, the expression was significantly attenuated for the dyslexia-linked allele as measured by allelic peak heights (*p* = 0.017 by two-tailed *t* test). To verify this initial analysis, we repeated the assay for all four dyslexic and four control participants by sequencing the SNP 6483T > A again. The results from five to six replicated sequencing assays for each participant are shown in Figure 2D. By the repeated measurements, the mean expression level of the dyslexia-associated allele in dyslexic participants was 66% of the same allele in controls (*p* < 0.0004 by two-tailed *t* test). To exclude the possibility that an individual SNP behaved aberrantly in the analysis, we also sequenced the SNP 6651T > A, yielding similar findings, and both SNP assays combined, the observation of allelic imbalance in cases versus controls was highly significant (*p* < 0.00005 by *t* test). As no SNP was specific for the *ROBO1* or *DUTT1* transcript only, we cannot assess isoform-specific down-regulation in the large family. In the translocation patient, two SNPs were heterozygous, both in the region corresponding to exons common to both *ROBO1* and *DUTT1* transcripts (6651T > A and 6923T > G). These SNPs revealed two alleles present in cDNA in the translocation patient, suggesting that *DUTT1* might be biallelically expressed even though the genomic structure of *ROBO1* was disrupted by translocation in one chromosome.

To study the possibility that the suppression of expression involved other genes than *ROBO1* in the dyslexia susceptibility haplotype, we genotyped known SNPs in the nearby genes *GBE1* (341C/G, 646A/G, 1597A/G, 1794C/T, 2349T/G, 2363A/G, 2761A/T) and *HTR1F* (528C/T, 783T/A) in the four dyslexic individuals of the large family (Figure 1C). Heterozygosity was detected for the *GBE1* SNPs 2363A > G and 646A > G in three patients. For these polymorphisms, normal biallelic expression was observed in all three patient samples in contrast to the finding with *ROBO1,* suggesting that transcription of *ROBO1* was specifically silenced. Heritable variation in allelic expression levels has previously been documented for several genes, and might conceivably arise by a number of different mechanisms, such as variation in enhancer and suppressor elements, splicing efficiency, transcript stability, or epigenetic modifications [24]. Two other positional candidate genes, *DRD3* and *5HT1F,* mapped outside the shared haplotype [17].

Seemingly silent exonic and intronic polymorphisms may induce disease related splice variants [25]. Thus, we studied the possibility of *ROBO1* alternative splicing by RT-PCR of all *ROBO1* and *DUTT1* exons from a dyslexic individual in the linkage family, an unrelated healthy control, and adult human brain cDNA. Seven novel splice variants were detected. Their cloning and sequencing revealed the exclusion of exons 2 (88 bp, 89–169 of *ROBO1*), 19 (27 bp, 2813–2829 of *ROBO1*), and 29 (196 bp 4745–4939 of *ROBO1*) entirely and exclusively of *DUTT1* exon 2 (346 bp 1019–1345 of *DUTT1*); the initial 165 bp of exon 22 (3037–3201 of *ROBO1*); 905 bp ranging from exons 24 to 28 (3603–4508 of *ROBO1*); and 878 bp ranging from exons 25 to 28 (3641–4528 of *ROBO1*) (Figure 1D). No splice variants were uniquely different between dyslexic and control individuals; however, quantitative differences could not be reliably assessed. Comparison of the genomic and cDNA sequences for *DUTT1* in several individuals suggested that exon 7 of *DUTT1* is not colinear with genomic sequence. Instead, *DUTT1* bases 1891–1900 (gttgggtct: valine, glycine, and serine), in the beginning of *DUTT1* exon 7 (*ROBO1* exon 8) belong to a new short exon, marked exon 7b (Figure 1D) corresponding to bases 5987–5995 of BAC RP11-588D3. These bases have previously been reported as part of the *DUTT1* gene but they are not included in the *ROBO1* cDNA sequence [23]. In all individuals sequenced, the cDNA sequence included the new exon 7b, indicating that it is included in the major splice form in at least brain and lymphoblast RNA.

As the known *ROBO1* sequence AF040990 starts from the transcription initiation site, we sought to determine the *ROBO1* 5′ UTR sequence by a BLAST search for expressed sequence tags homologous for the 5′ *ROBO1* region. The expressed sequence tag AW450262, homologous to *ROBO1* exons 1 and 2, indicated an additional site (referred to as *ROBO1* exon a) upstream on BAC AC125624 (bases 28508–28470) (Figure 1D). RT-PCR with an initial primer in the exon a sequence and primers in *ROBO1* exons 1 and 4 revealed an additional 52 bp (bases 34119–34168 of BAC AC125815) 5′ of the transcription initiation site on *ROBO* exon 1 and also confirmed the presence of the novel exon a on the BAC AC125624. Additional primers were designed 5′ to the novel exon a, and RT-PCR performed similarly as above showed the a exon to span at least 129 bp (bases 28593–28466 of BAC AC125624). 5′ rapid amplification of cDNA ends (RACE) revealed additional 326 bps, stretching the exon to 28919–28466 on the BAC AC125624.

**Figure 3:**
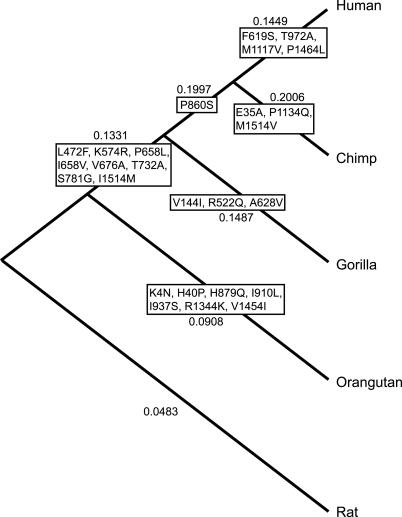
Coding Changes of *ROBO1* during Primate Evolution Phylogenetic tree of ROBO1 protein evolution in hominoids. Rat was used as the out-group in sequence comparisons. dN/dS ratios of the branches were calculated with the Codeml program, assuming a freely varying ratio. A model in which omega value was higher in lineages leading to humans, chimpanzees, and gorillas was significantly better than a free-ratio model (*p* < 0.001).

A significant fraction of human genes has been under positive Darwinian selection since the common ancestor of humans and chimpanzees [26]. For example, *FOXP2,* the gene implicated in speech and language, has undergone a selective sweep during human evolution [27]. Therefore, we sequenced *ROBO1* from chimpanzee, pygmy chimpanzee, gorilla, and orangutan, and used the rat *ROBO1* sequence as the out-group. We used likelihood ratio test to analyze variation in the selective pressure in *ROBO1* sequence in the different lineages. Non-synonymous and synonymous (dN and dS) ratio was smaller than 1 in all lineages, implicating purifying Darwinian selection. However, the likelihood ratio test rejected the null hypothesis of fixed dN/dS ratio in all lineages. A model in which omega value was higher in lineages leading to humans, chimpanzees, and gorillas was significantly better than a free-ratio model (*p* < 0.001) (Figure 3 and Tables S1 and S2). This suggests that the selective pressure for *ROBO1* gene has changed 12 to16 million years ago, after the divergence of the orangutan branch.

## Discussion

Our data suggested that two functional copies of *ROBO1* are required in brain development to acquire normal reading ability, and partial haplo-insufficiency for *ROBO1* may predispose humans to specific dyslexia. *robo* was originally identified in *Drosophila* in a search for genes controlling the midline crossing of axons; in mutant *robo* embryos, axons cross and recross across the midline too many times [20–22]. The human ortholog of *robo*, *ROBO1* (also named *DUTT1*), was identified as a potential tumor-suppressor gene in a small-cell lung cancer cell line [28]. *ROBO1* and *DUTT1* are presumed to be alternative splice variants with different initial exons and initiation codons and may thus have in part distinct functions [23]. Homozygous *Robo1/Dutt1* knockout mice are embryonically lethal, but heterozygous mice were found to develop lymphomas and lung adenocarcinomas at high frequency [29]. The human *ROBO1/DUTT1* locus has been found deleted in a child with developmental delay and congenital anomalies but without cancers [30]. These observations seem to pose a dilemma for understanding the functions of *ROBO1* in different species.

*robo* encodes a transmembrane receptor that belongs to the immunoglobulin superfamily. It consists of immunoglobulin domains, three fibronectin domains, a transmembrane domain, and a long intracellular region with no recognizable motifs, but four proline rich repeats, which are suggested to act with *enabled, abelson,* SH3 binding proteins, and other downstream signaling molecules [20,31]. As shown by our data, *ROBO1* undergoes alternative splicing in a more complex manner than previously appreciated and the functions of these alternative splice variants are unknown. It is possible that still different promoters and splice variants in different tissues cause alternative phenotypes. The translocation in our patient disrupts specifically *ROBO1,* but not *DUTT1,* and therefore does not contradict the previous observations of *DUTT1* as a tumor suppressor gene. We thus hypothesize that the two-splice variants in humans are associated with different key functions in different tissues: *ROBO1* might correspond more closely to functions in the human brain that are modeled by neuronal functions in the fruit fly, and *DUTT1* functions appear to correspond to the mouse model of lung tumorigenesis.

In the fruit fly, robo is a receptor for secreted repellent slit proteins and acts as a gatekeeper of axonal crossing on the left-right axis. Activation of robo makes axons indifferent to the chemoattractant netrin, a ligand of the DCC receptor [32]. Netrin-dependent activation of the DCC receptor increases transient phosphorylation of ERK1, ERK2, and the transcription factor ELK1. The activation of the ERK pathway has been suggested as necessary for experience-dependent plasticity and for long-term potentiation of synaptic transmission in visual cortex development in the rat [33]. Interestingly, a possible ELK1 binding site was altered and associated with dyslexia in *DYX1C1,* a candidate gene for dyslexia susceptibility on Chromosome 15q21, and ELK1 has been implicated in learning in the rat [13,34–36]. A suggested functional role for *DYX1C1* remains unconfirmed, because its associations have not been unambiguously replicated, leaving the relevance of the ELK1 binding site open [37–42]. Slit/Robo/DCC signaling has also been implicated in cortical dendritic guidance and development [43,44]. Furthermore, a recently identified dyslexia susceptibility locus on Xq27.3 includes the *SLITRK2* and *SLITRK4* genes, which belong to the SLIT and NTRK-like family of genes involved in mouse neurite outgrowth and show high homology to Slit proteins [10,45,46]. Thus, the identification of *ROBO1* as a susceptibility gene in dyslexia may implicate a key developmental pathway in which slight disturbances may lead to specific reading disability.

Genomic sequences and predicted transcripts for *ROBO1* in four apes revealed a high level of variation between the related species and humans (Figure 3). We detected seven amino acid changes between human and chimpanzee and 20 between humans and orangutan. An analysis of dN/dS substitutions revealed that the selective pressure on *ROBO1* has changed after the divergence of the orangutan branch. Although according to stringent criteria, only dN/dS ratios higher than 1 are regarded as signs of positive selection; it has been shown that genes expressed in the brain are under stronger selective pressure than genes expressed, for example, in the liver [47]. In addition, in primates many brain-expressed genes show significantly higher dN/dS ratios than housekeeping genes when compared to the rodent counterparts, even though the dN/dS ratios in primates are still well below 1. Interestingly, it was recently shown that the evolution of *SLIT1,* a ligand for *ROBO1,* has been significantly faster in primates than in rodents [48]. Also other proteins involved in axonal path-finding, such as SEMA4F and EPHA6, were shown to have undergone adaptive evolution [48]. We propose that *ROBO1* might have undergone rapid changes in the recent primate evolution that may be related to its largely uncharacterized functions in the human brain.

Taken together, these results implicate a well-known pathway of neuronal development in a highly specific cognitive function in humans. This function attributed here to the *ROBO1* transcript variant including newly discovered exons is distinct from the role that has been suggested in lung tumorigenesis for the alternatively spliced transcript variant *DUTT1.* This insight may open new visions for understanding complex brain processes and provide a framework for building testable hypotheses for the biology of reading.

## Materials and Methods

### Patients.

A multiplex four-generation family with severe dyslexia segregating in a dominant fashion (Figure 2A) has been previously studied for genetic linkage and phenotype [17,19]. Dyslexia was diagnosed in 27 out of 74 family members by thorough testing including an intelligence test, a Finnish reading and writing test for adults and for children according to their school grade, and a neuropsychological test battery [49–52]. Detailed psychological evaluation of this family has been reported elsewhere [19].

A dyslexic individual with a balanced reciprocal translocation t (3;8) (p12;q11) came to our attention because of infertility and was diagnosed with oligoteratozoospermia. He has three siblings, and all four children have been neuropsychologically evaluated at a specialist hospital. However, the family members were not available for retesting and thus our data are based on the clinical records. The translocation carrier and his sister were diagnosed with severe dyslexia while the other two siblings had subnormal intelligence, but not dyslexia. The mother was reported as a good reader, but no information on reading performance was available on the deceased father. The other three siblings have a normal karyotype, whereas the parents were not available for karyotyping. Because the index case presented with infertility, but no such history or miscarriages were recorded for his mother, it is likely that the translocation had arisen de novo. Thus, the translocation and dyslexia did not apparently cosegregate in two siblings, but as dyslexia is a complex phenotype, no inference is conclusive to either reject or confirm a possible causal association of the translocation with dyslexia in the index case.

For association studies, dyslexic and non-dyslexic individuals were recruited from 23 unrelated families and 33 unrelated dyslexic and non-dyslexic couples from the Department of Pediatric Neurology at the Hospital for Children and Adolescents, University of Helsinki, and the Child Research Centre, Jyväskylä, Finland. Additional population controls consisted of 100 anonymous blood donors. The diagnosis and degree of dyslexia were determined by Finnish reading and spelling tests designed for children and adults [49,50]. Intelligence was estimated by Wechsler tests for adults (WAIS-R) or for children (WISC-R) [51,52]. The diagnostic criteria for dyslexia included normal performance intelligence quotient (PIQ > 85) and remarkable deviation (depending on age, at least two years) in reading skills. This study has been approved by the ethical review board of the Helsinki University Central Hospital, and informed consent was obtained from the participants.

### Fluorescence in situ and Southern hybridization.

YAC clones A136E9 (Washington University, St. Louis, Missouri, United States), 34FC9, 15AC10, 39F13, 2DG9, 25DH8, 35AH8 (ICI/Zeneca), 912A11, 934E8, 422A6, 959F5, 650C2, 938D4, and BAC RP11-143B12 were used as probes in fluorescence in situ hybridization experiments. The genomic sequence of clone RP11-143B12 was obtained by BLAST search with the BAC 5′ and 3′ends (AQ373182, T7, and AQ373179 Sp6, respectively) to a Chromosome 3 scaffold sequence on the Celera public database (http://public.celera.com/cds/login.cfm) and to BACs in the National Center for Biotechnology CBI database (http://www.ncbi.nlm.nih.gov/).

Southern hybridization probes were PCR-amplified genomic fragments from non-repetitive regions on the BAC clone RP11-143B12. The repeats in the clone sequence were detected by RepeatMasker (http://repeatmasker.genome.washington.edu/cgi-bin/RepeatMasker) and PCR primers were designed by Primer3 (http://frodo.wi.mit.edu/cgi-bin/primer3/primer3_www.cgi) to encompass non-repetitive segments of 700–1,000 bp (primer sequences available from authors on request). PCR assays were performed under standard conditions and 10 ng of the purified PCR products (Qiagen PCR purification kit) (Qiagen, Valencia, California, United States) and 10 ng of the purified probes were labeled with [a-32P] dCTP (Rediprime DNA Labeling System, Amersham Biosciences, Little Chalfont, United Kingdom). Southern blotting and hybridizations were performed by standard protocols with seven μg of DNA from the translocation patient and a healthy control individual digested in separate reactions with BamHI, BglII, EarI, EcoRI, HaeII, HindIII, NcoI, and PstI (New England Biolabs, Beverly, Massachusetts, United States).

### Polymorphism screening of *ROBO1* and expression analysis.

All *ROBO1* and *DUTT1* exons were PCR-amplified and sequenced from genomic DNA and the cDNA of selected individuals from the large linkage pedigree (Figure 2A). In addition, the novel exonic sequences were identified and 2 kb of *ROBO1* promoter region upstream of the novel exon a and the 3′ UTR of *ROBO1* variant 2 were sequenced. BACs corresponding to exons were identified through BLAST searches. Primate DNA samples were obtained from the Coriell Institute (Camden, New Jersey, United States) (Primate Panel PRP00001) and orthologs of *ROBO1* were sequenced directly after PCR with human-specific primers.

RNA was extracted from EBV transformed lymphocyte cell lines from four dyslexic and four normal readers by Ficoll gradient centrifugation (Qiagen Rneasy purification kit) and RT-PCR was used to amplify cDNA segments containing heterozygous SNPs in genomic DNA. As controls, we used genomic DNA samples from the same individuals as well as brain mRNA (Clontech, Palo Alto, California, United States). All sequencing was performed using dye-terminator chemistry and automated sequencers (ABI, Columbia, Maryland, United States).

To assess the allele-specific expression, we followed a standard method [53]. The assay is based on the comparison of allelic peak heights (in arbitrary units) in cDNA sequence (after RT-PCR) and genomic sequence from each individual. An allelic ratio is calculated for each sequence (e.g., height of allele A per height of allele C). Because the allelic ratio in genomic sequence is by definition 1 (each allele is present as one copy per diploid genome), but the actual value may differ from 1 (because of chemical properties of the sequencing reactions), the cDNA allelic ratio values are normalized by dividing by the genomic allelic ratio in each experiment. To assess whether the normalized cDNA allelic ratios differed in dyslexic patients as compared with controls, the values from replicated experiments were compared between the groups by two-tailed *t*-test. To estimate the degree of attenuation of one allele in dyslexic patients, the average cDNA allelic ratio in dyslexic patients was divided by the average cDNA allelic ratio in controls. Standard deviation of the measurements was calculated on replicated experiments.

### 5′ RACE.

5′ RACE was performed on human brain RNA using the SMART RACE cDNA Amplification kit (Clontech). 5′ cDNA ends were amplified with the Universal Primer A and a specific Robo 5′ RACE-R1 (gcagacgcagccctgcaacttt) primer, followed by nested PCR with the Nested Universal Primer A. PCR products were purified and directly sequenced (ABI).

### Evolutionary analysis of *ROBO1* sequence*.*

Likelihood ratio test was performed with the Codeml program of the PAML package [26].

## Supporting Information

Table S1 Comparison of *ROBO1* between Human and Four Non-Human Primates Nucleic acid (according to AF040990 exons 1 to 29, NM_133631 exon 30) and amino acid changes are shown for each exon of *ROBO1* in comparison to the corresponding human BAC sequence; + indicates the presence of a change in a non-human species. Amino acid changes are shaded. No differences were observed for *DUTT1* exon 1.
(311 KB DOC)

Table S2 Analysis of ROBO1 Evolution with PAML Likelihood values and parameter estimates under different models
(19 KB XLS)

### Accession Numbers

The GenBank (http://www.ncbi.nlm.nih.gov/geo) accession numbers for genes discussed in this paper are *DUTT1* (Z95705), *GBEI* (NM_000158), *HTRIF* (NM_000866), human homolog 1of the *Drosophila roundabout gene, ROBO1* (AF040990), the first intron of *ROBO1* (NM_002941), *ROBO1* variant 2 (NM_133631), Rat *ROBO1* (NM_022188), *Homo sapiens* clone sequences for BACs RP11-588D3 (AC055731) and BAC RP11-26M20 (AC106720).

## Abbreviations

BAC: bacterial artificial chromosome
bp: base pair
dN/dS: non-synonymous and synonymous
kb: kilobase
RACE: rapid amplification of cDNA ends
SNP: single nucleotide polymorphism
