# Supplementary material for: The Axon Guidance Receptor Gene ROBO1 Is a Candidate Gene for Developmental Dyslexia
Source: PLoS Genet. 2005 Oct 28;1(4):e50. doi: 10.1371/journal.pgen.0010050 (PMC1270007; doi:10.1371/journal.pgen.0010050)
Supplement: Table S1 — Nucleic acid (according to AF040990 exons 1 to 29, NM_133631 exon 30) and amino acid changes are shown for each exon of ROBO1 in comparison to the corresponding human BAC sequence; + indicates the presence of a change in a non-human species. Amino acid changes are shaded. No differences were observed for DUTT1 exon 1. (311 KB DOC) [file pgen.0010050.st001.doc]

Table 1. Comparison of *ROBO1* between human and four nonhuman primates. Nucleic acid (according to AF040990 exons 1 to 29, NM_133631 exon 30) and aminoacid changes are shown for each exon of *ROBO1* in comparison to the corresponding human BAC sequence; + indicates the presence of a change in a nonhuman species. Amino acid changes are shaded. No differences were observed for *DUTT1* exon 1.

| Exon | Nucleic acid change human>primate | Amino acid change human>primate | Pygmy chimpanzee | Chimpanzee | Gorilla | Orangutan |
| --- | --- | --- | --- | --- | --- | --- |
| 1 | 12A>C | K4>N |  |  |  | + |
| 2 | 104A>C | E35>A |  | + |  |  |
| 2 | 119A>C | H40>P |  |  |  | + |
| 3 | 222T>G | P74>P |  |  |  | + |
| 3 | 255A>C | A85>A |  | + |  |  |
| 3 | 273T>C | A91>A |  |  |  | + |
| 3 | 276A>G | E92>E |  |  | + |  |
| 3 | 342C>T | P115>L |  |  |  | + |
| 3 | 369G>A | P123>P |  |  |  | + |
| 3 | 430G>A | V144>I |  |  | + |  |
| 4 | 576A>G | Q192>Q |  |  |  | + |
| 4 | 579T>C | P193>P |  |  |  | + |
| 4 | 594T>G | P198>P |  |  |  | + |
| 4 | 603C>A | T201>T |  |  | + |  |
| 5 | none |  |  |  |  |  |
| 6 | none |  |  |  |  |  |
| 7 | 963A>G | T318>T |  |  |  | + |
| 7 | 1041T>G | V344>V | + | + |  |  |
| 8 | 1089T>C | A363>A |  |  |  | + |
| 8 | 1149C>T | F383>F | + | + | + | + |
| 9 | 1323A>G | A441>A |  |  | + |  |
| 10 | 1365A>G | P455>P |  |  |  | + |
| 10 | 1401C>T | A467>A | + | + | + | + |
| 10 | 1416C>A | F472>L |  |  |  | + |
| 10 | 1452C>T | P484>P |  |  |  | + |
| 10 | 1513T>C | L505>L |  |  |  | + |
| 10 | 1521T>C | N507>N |  |  |  | + |
| 10 | 1527A>G | V509>V |  |  |  | + |
| 11 | 1565G>A | R522>Q |  |  | + |  |
| 12 | 1662T>C | P554>P |  |  |  | + |
| 12 | 1707G>T | V569>V |  |  |  | + |
| 12 | 1710A>G | T570>T |  |  |  | + |
| 12 | 1721G>A | R574>K |  |  |  | + |
| 13 | 1856C>T | S619>F | + | + | + | + |
| 13 | 1883C>T | A628>V |  |  | + |  |
| 14 | 1971C>T | V657>V |  |  |  | + |
| 14 | 1973T>C | L658>P |  |  |  | + |
| 14 | 2022A>G | G674>G |  | + | + | + |
| 14 | 2027C>T | A676>V |  |  |  | + |
| 14 | 2049C>T | P683>P |  |  |  | + |
| 14 | 2053G>A | V685>I |  |  |  | + |
| 15 | 2190G>A | T730>T |  |  |  | + |
| 15 | 2149G>A | A732>T |  |  |  | + |
| 15 | 2211A>T | V737>V | + | + | + | + |
| 15 | 2214C>T | I738>I |  |  | + |  |
| 15 | 2301T>C | F767>F | + | + | + | + |
| 16 | 2341G>A | G781>S |  |  |  | + |
| 17 | 2574T>A | A858>A |  |  |  | + |
| 17 | 2588T>C | S860>P |  |  | + | + |
| 17 | 2586A>G | V862>V |  |  |  | + |
| 18 | 2622T>A | H874>Q |  |  |  | + |
| 18 | 2679G>C | V893>V |  |  |  | + |
| 18 | 2691G>T | P897>P |  |  |  | + |
| 18 | 2709T>C | I903>I |  |  | + | + |
| 18 | 2728A>C | I910>L |  |  |  | + |
| 18 | 3733C>T | L911>L |  |  |  | + |
| 18 | 2757T>C | Y919>Y |  |  |  | + |
| 18 | 2784T>G | L928>L |  |  | + |  |
| 18 | 2793C>T>A | T931>T | + | + | + | + |
| 18 | 2796C>T | Y932>Y |  |  |  | + |
| 19 | none |  |  |  |  |  |
| 20 | none |  |  |  |  |  |
| 21 | 2886T>C | P962>P | + | + | + |  |
| 21 | 2889A>T | G963>G |  |  |  | + |
| 21 | 2900T>G | I967>S |  |  |  | + |
| 21 | 2914G>A | A972>T | + | + | + | + |
| 21 | 2926C>T | L976>L |  |  |  | + |
| 21 | 2931A>G | A977>A | + | + |  | + |
| 21 | 2946T>C | N982>N |  |  |  | + |
| 21 | 2997T>C | N999>N |  |  |  | + |
| 21 | 3021T>G | T1007>T |  |  |  | + |
| 21 | 3033C>T | R1011>R |  |  |  | + |
| 22 | 3219C>T | Y1073>Y | + | + |  | + |
| 22 | 3303A>G | P1101>P |  |  |  | + |
| 22 | 3349G>A | V1117>M | + | + | + | + |
| 23 | 3401C>A | P1134>Q | + | + |  |  |
| 24 | 3567C>A | P1189>P |  |  | + |  |
| 25 | 3726T>C | P1242>P | + | + |  |  |
| 25 | 3780T>G | T1260>T |  |  |  | + |
| 25 | 3840G>A | E1280>E |  |  |  | + |
| 26 | 3915C>T | I1305>I |  |  |  | + |
| 26 | 3996C>T | D1332>D |  |  |  | + |
| 26 | 4002C>T | A1334>A |  |  | + | + |
| 26 | 4031G>A | E1337>E |  |  |  | + |
| 26 | 4074T>C | S1358>S |  |  |  | + |
| 26 | 4101G>A | T1367>T |  |  |  | + |
| 26 | 4155C>T | S1385>S |  |  |  | + |
| 26 | 4200T>C | D1400>D |  |  |  | + |
| 26 | 4206C>T | D1402>D |  |  |  | + |
| 26 | 4221C>T | V1407>V | + |  |  |  |
| 26 | 4230G>A | A1410>A |  |  |  | + |
| 26 | 4254A>G | V1418>V | + | + | + | + |
| 27 | 4302G>A | A1434>A |  | + |  |  |
| 27 | 4329C>T | P1443>P | + | + | + | + |
| 27 | 4356C>T | A1452>A |  |  |  | + |
| 27 | 4359C>T | A1453>A |  |  |  | + |
| 27 | 4360G>A | V1454>I |  |  |  | + |
| 27 | 4391T>C | L1464>P | + | + | + | + |
| 28 | 4540A>G | M1514>V | + | + |  |  |
| 28 | 4542G>A | M1514>I |  |  |  | + |
| 28 | 4569C>T | S1521>S |  |  |  | + |
| 28 | 4602G>A | V1534>V | + | + | + | + |
| 28 | 4617G>A | Q1539>Q |  |  |  | + |
| 29 | none |  |  |  |  |  |
| 30 | 5840-42 CAT | 3’UTR | + | + | - | - |
| 30 | 5863A>G | 3’UTR |  |  |  | + |
| 30 | 5889G>A | 3’UTR |  |  |  | + |
| 30 | 5905G>A | 3’UTR | + |  |  |  |
| 30 | 5940A>G | 3’UTR | + | + | + | + |
| 30 | 6017insC | 3’UTR |  | + |  |  |
| 30 | 6040T>C | 3’UTR | + | + | + | + |
| 30 | 6058C>G | 3’UTR |  |  |  | + |
| 30 | 6105C>A | 3’UTR |  |  | + |  |
| 30 | 6112G>C | 3’UTR |  |  |  | + |
| 30 | 6130G>C | 3’UTR | + | + | + | + |
| 30 | 6174A>G | 3’UTR |  |  |  | + |
| 30 | 6180A>C | 3’UTR | + | + |  |  |
| 30 | 6200G>A | 3’UTR |  |  | + |  |
| 30 | 6203-05delGAT | 3’UTR | + | + | + | + |
| 30 | 6216insTGC | 3’UTR | + | + | + | + |
| 30 | 6243insTTCT | 3’UTR | + | + |  |  |
| 30 | 6249C>T | 3’UTR | + | + |  |  |
| 30 | 6352G>A | 3’UTR |  |  | + |  |
| 30 | 6384T>A | 3’UTR |  |  |  | + |
| 30 | 6428G>C | 3’UTR |  |  |  | + |
| 30 | 6429G>T | 3’UTR | + | + | + | + |
| 30 | 6539T>C | 3’UTR | + | + | + | + |
| 30 | 6565-6567 delTCA | 3’UTR | + | + | + | + |
| 30 | 6573T>C | 3’UTR |  |  |  | + |
| 30 | 6619delA | 3’UTR |  |  |  | + |
| 30 | 6624G>A | 3’UTR |  |  | + |  |
| 30 | 6651T>A | 3’UTR |  |  | + |  |
| 30 | 6751polyCT | 3’UTR | + |  |  | + |
| 30 | 6851G>T | 3’UTR |  |  | + |  |
| 30 | 6967T>C | 3’UTR | + | + |  |  |
| 30 | 6995A>G | 3’UTR |  |  | + |  |
| 30 | 6996insAG | 3’UTR | + |  |  |  |
| 30 | 7021C>G | 3’UTR |  |  |  | + |
| 30 | 7027C>T | 3’UTR | + | + |  | + |
| 30 | 7029A>C | 3’UTR |  | + |  |  |
| 30 | 7056A>G | 3’UTR |  |  |  | + |
| 30 | 7078A>C | 3’UTR | + | + | + | + |
| 30 | 7190 A>G | 3’UTR |  |  | + |  |
| 30 | 7196 del ATAA | 3’UTR | + | + | + | + |
| 30 | 7385 C>T | 3’UTR | + | + | + | + |
| 30 | 7438 A>T | 3’UTR | + |  |  |  |
